# The use of a DBT skills-based group in aiding with the inpatient and outpatient treatment demands of a specialized eating disorders unit

**DOI:** 10.1186/2050-2974-1-S1-O6

**Published:** 2013-11-14

**Authors:** Lyndsey Nolan, Sarah Mitchell

**Affiliations:** 1 Eating Disorders Unit, NorthWestern Mental Health, Royal Melbourne Hospital, Australia

## 

The Royal Melbourne Hospital provides inpatient, outpatient and day patient treatment to over 130 patients within the NorthWestern catchment area of Victoria each year. The RMH Eating Disorders Unit is the largest public inpatient service Room: State-wide offering 8 inpatient beds, with an average length of stay of 36 days. The severity, chronicity and complexity of emerging presentations has resulted in a greater demand for inpatient beds, exhausted wait lists, and placed further need and demand on outpatient and transitional support. The Dialectical Behavioural Therapy (DBT) group for Eating Disorders was developed and introduced to the Unit in mid-2011 as a means to provide support, treatment and relapse prevention to those patients who presented with borderline personality disorder (BPD) features and/or often required multiple admissions.

The DBT group is a skills-based closed group, which runs for 2.5 hours weekly for 15 weeks. The group focuses on psycho-education of eating disorders, mindfulness, emotional regulation, distress tolerance, and interpersonal effectiveness. Each cohort is evaluated using pre-test and post-test measures as well as weekly participation feedback. The outcome measures to date have shown that the DBT outpatient group has been effective in reducing BPD features and impulsive behaviours; created positive shifts in mood, unhelpful thought patterns and schemas; reduced stress and anxiety; and attributed to lower readmission rates.

This abstract was presented in the **Adult Treatment and Services** stream of the 2013 ANZAED Conference.

